# The complex nature of heterogeneity and its roles in breast cancer biology and therapeutic responsiveness

**DOI:** 10.3389/fendo.2023.1083048

**Published:** 2023-02-23

**Authors:** Karla Andrade de Oliveira, Surojeet Sengupta, Anil Kumar Yadav, Robert Clarke

**Affiliations:** ^1^ The Hormel Institute, University of Minnesota, Austin, MN, United States; ^2^ Department of Biochemistry and Pharmacology, Universidade Federal do Piaui, Piauí, Brazil

**Keywords:** heterogeneity, endocrine therapy, drug resistance, breast cancer, systems biology

## Abstract

Heterogeneity is a complex feature of cells and tissues with many interacting components. Depending on the nature of the research context, interacting features of cellular, drug response, genetic, molecular, spatial, temporal, and vascular heterogeneity may be present. We describe the various forms of heterogeneity with examples of their interactions and how they play a role in affecting cellular phenotype and drug responses in breast cancer. While cellular heterogeneity may be the most widely described and invoked, many forms of heterogeneity are evident within the tumor microenvironment and affect responses to the endocrine and cytotoxic drugs widely used in standard clinical care. Drug response heterogeneity is a critical determinant of clinical response and curative potential and also is multifaceted when encountered. The interactive nature of some forms of heterogeneity is readily apparent. For example, the process of metastasis has the properties of both temporal and spatial heterogeneity within the host, whereas each individual metastatic deposit may exhibit cellular, genetic, molecular, and vascular heterogeneity. This review describes the many forms of heterogeneity, their integrated activities, and offers some insights into how heterogeneity may be understood and studied in the future.

## Introduction

Endocrine resistance is a major clinical problem for the treatment of hormone-receptor (HR+) positive breast cancer (BC). HR+ tumors comprise the most prevalent molecular subtype, representing over 70% of all newly diagnosed breast cancers each year. However, using endocrine monotherapies to improve the overall survival (OS) rates for patients with HR+ breast cancer has shown only modest further increases since the introduction of tamoxifen in the early 1970s. As monotherapies, only Fulvestrant has shown an ability to increase OS; aromatase inhibitors confer no OS benefit relative to tamoxifen (1–3). Adding a CDK4,6 inhibitor such as abemaciclib or ribociclib to any of the current endocrine therapies improves OS beyond that of the endocrine monotherapy (4–6). Nonetheless, many patients experience a recurrence of their HR+ breast cancer, often many years after completing an apparently successful treatment regimen (7). Recurrence is almost always fatal, with the predicted number of breast cancer deaths from all molecular subtypes in the United States alone expected to exceed 42,000 in 2023 (8).

Endocrine therapies that target the activation state of estrogen receptor-α (ER; ESR1) fall into two broadly defined agent classes – antiestrogens and aromatase inhibitors. Antiestrogens act primarily by competing with endogenous 17β-estradiol and other estrogens for binding to ER and blocking its activation. Tamoxifen and structurally related triphenylethylene-like compounds are mostly partial agonists and often described as selective estrogen receptor modulators (SERMs). The ER antagonist properties of tamoxifen are evident in its ability to reduce the risk of breast cancer mortality by almost one-third (7), whereas its agonist properties are largely responsible for the increased risk of endometrial cancer associated with long term use (9). Fulvestrant and related compounds also compete with estrogens for ER binding but can target the receptor protein for degradation and are often referred to as selective estrogen receptor downregulators or degraders (SERDs). The mechanistic importance of ER degradation as a driver of antineoplastic activity, relative to competing for estrogen binding to ER, may be minor (10, 11). Both SERDs and SERMS occupy the ligand binding domain of ER and induce a conformation in the protein that alters the ability of ER to act as a transcription regulator. Aromatase inhibitors act by blocking the enzyme activity of the aromatase protein that converts androgens to estrogens, depriving ER of its activating ligands. Hence, the ER protein remains as a largely inactive transcriptional regulator unless modified by selective mutation or phosphorylation.

Two ligand independent actions can alter ER function – mutation or phosphorylation of specific residues. ER can be phosphorylated at multiple residues, with serine-167 (S-167) and serine-118 (S-118) in the AF1 region of the ER protein being the most prevalent (12). Higher expression of either pS-167 and/or pS-118 is mostly associated with favorable clinical outcome in patients treated with tamoxifen (13–16). Increased expression of p-S118 in secondary breast tumors post relapse may be predictive of longer survival (17). The presence of ESR1 mutations following failure on an aromatase inhibitor-based regimen is widely reported, with 18%-55% of recurrent tumors expressing one or more ER mutations (18–21). Two sites, D538 and Y537, account for most of these ER mutations. Meta-analysis of the effects of ESR1 mutations in breast cancer revealed that D538G is the most frequently detected mutation and is associated with poor relapse free survival. Most of the ESR1 mutations detected were associated with aromatase inhibitor treatment (22). Since the prevalent ESR1 mutations encode a constitutively active ER protein, these mutations appear to drive up to ~40% of aromatase inhibitor resistant tumors. Drivers of the remaining 50-60% of these aromatase inhibitor resistant tumors remain to be discovered. In clinical studies, the benefit of antiestrogen therapy does not appear to be substantially affected by the presence of these ER mutations (23, 24). Unlike aromatase inhibitor therapies, recurrence following an antiestrogen therapy is associated with a relatively small increase in the prevalence of ER mutations (22).

Whether by loss of access to natural ligands as occurs with aromatase inhibition, or as a consequence of binding an antiestrogen, altered ER function directly affects cell fate by compromising two major cell functions – survival and proliferation. The most readily apparent of these changes is an often rapid slowing of proliferation, usually a result of cells accumulating in the G_1_, or exiting to the G_0_, phase of the cell cycle. Cell death occurs more slowly. Both changes are observed in endocrine sensitive, HR+ experimental models and in many HR+ breast tumors in patients. For example, in endocrine treated breast tumors reduced proliferation is evident from the drop in expression of the Ki67 proliferation marker (25, 26), whereas tumor shrinkage reflects increased cell death. Not surprisingly, these changes occur on different time scales, with changes in Ki67 detected within days and tumor shrinkage often occurring over many weeks or months (26, 27). Unlike the rapid induction of cell death that can occur with chemotherapy and often leads to a complete pathological response (pCR) that predicts a good clinical response, the much slower induction of cell death and tumor shrinkage that accompany endocrine therapies rarely lead to a rapid pCR (28, 29). Nonetheless, both cytotoxic and endocrine interventions confer a broadly comparable increase in OS (28, 30), most likely reflecting the ability of each modality to increase the rate of breast cancer cell death within tumors.

The time scales for cell death and tumor shrinkage are likely further affected by the cell populations targeted by each type of intervention. For example, cytotoxic chemotherapy may kill proliferating infiltrating immune cells and cancer associated fibroblasts (CAFs) in the tumor microenvironment (TME) that are often ER-negative and likely not killed by endocrine therapies. While cellular heterogeneity can be one determinant of responsiveness to any given therapy, heterogeneity is complex and multifaceted.

## The complex nature of heterogeneity

**Figure 1:**
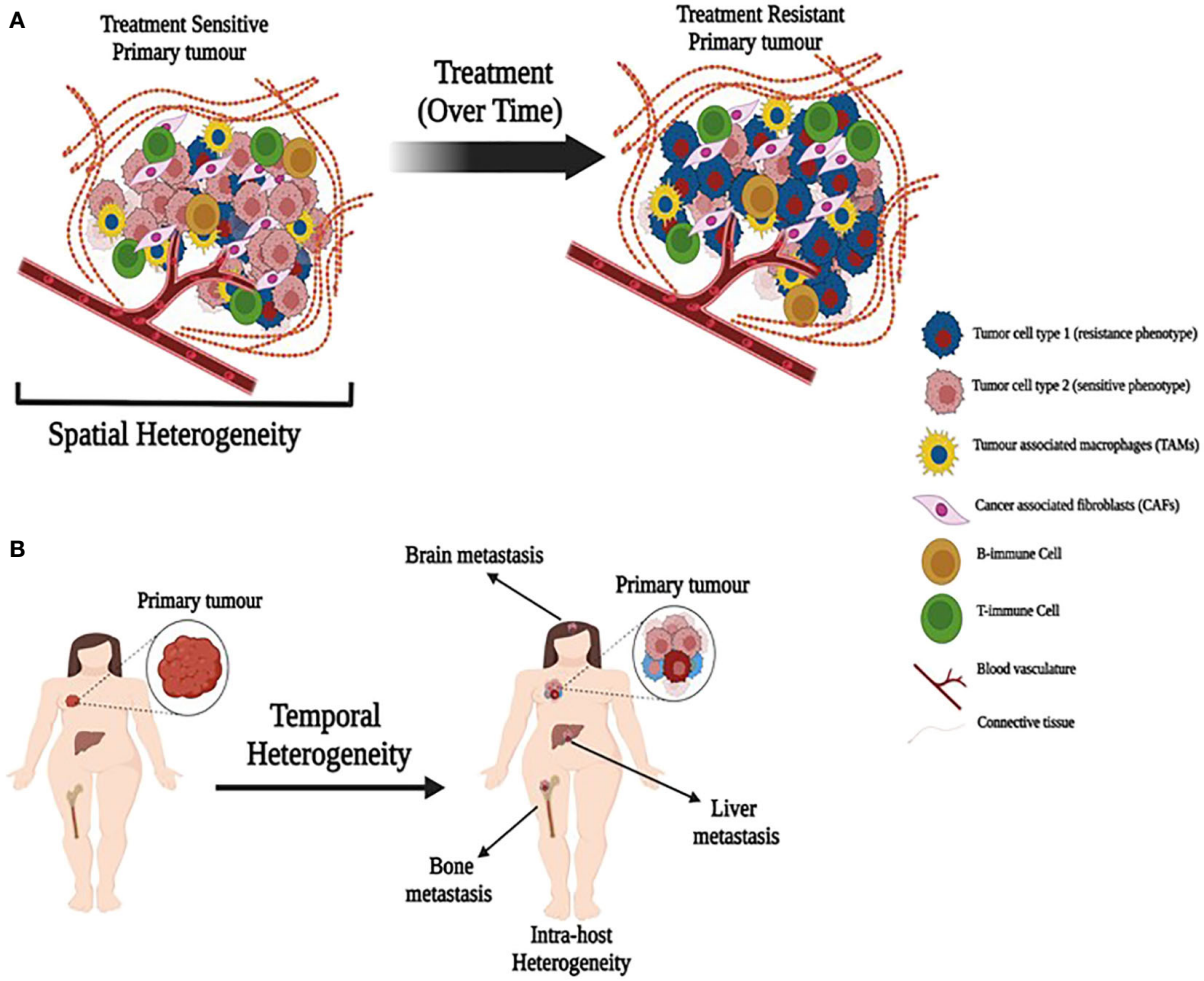
Illustration depicting spatial and temporal heterogeneity. **(A)** A primary tumor with heterogeneous population of treatment sensitive and resistant cancer cells along with tumor associated cells. Treatment over time may allow expansion of resistant cells. **(B)** Primary tumors metastasize over time to produce metastatic tumors in different organs with different cell composition (temporal heterogeneity) that may also lead to intra-host heterogeneity.

Breast tumors and their associated microenvironments are often described as being highly heterogeneous. Generally, this is taken to reflect cellular heterogeneity, although the precise nature of the heterogeneity invoked may not be defined explicitly. Cellular heterogeneity usually refers to the presence of different cell types within the TME and exhibits both spatial and temporal variability within an individual tumor and among different colonized sites in the same patient (primary tumor and distant metastatic sites). While tumor cells may remodel some features of their microenvironment to create a supportive niche at a site distant from the primary tumor, the microenvironment of each metastasis will reflect, to some degree, the normal tissue in which it resides. In breast cancer, common metastatic sites include bone, brain, lung, lymph nodes, skin, and viscera. Location in the body creates one form of spatial heterogeneity (**Figure 1A**). Each metastasis arises over time, conferring a form of temporal heterogeneity (**Figure 1B**).

Cellular heterogeneity reflects the presence of cancer cells, normal and/or stromal cells such as non-transformed adipocyte, epithelial, myoepithelial, fibroblast and myofibroblast cells, and infiltrating immune cells including various T-cells, macrophages, dendritic and natural killer (NK) cells. As definitively normal cells, their genome sequence profiles would be expected to be similar. However, each of these cell types has a different phenotype and function and will also express different transcriptome, proteome, metabolome and epigenome profiles (molecular heterogeneity). These various profiles also exhibit dynamism (creating another form of temporal heterogeneity) as each cell type responds to the changing extrinsic signals it receives and the intrinsic responses and functions it performs in response. For example, macrophages in the TME may exist in different states that can be M1-like (pro-inflammatory, anti-tumorigenic) or M2-like (anti-inflammatory, pro-tumorigenic) (31, 32). Hence, there is significant spatiotemporal molecular heterogeneity in the TME, reflecting the location of each cell type and its immediate neighbors and their respective interactions and cell states.

The tumor cell compartment of the TME also exhibits spatiotemporal heterogeneity in its genome, epigenome, transcriptome, and proteome profiles. The genetic heterogeneity from acquired mutations is inherent in cancer, with different cancer cells exhibiting some features of their genetic ancestry in a manner that allows investigators to follow the genetic evolution of the cancer cells in a tumor (33). These various features of heterogeneity are affected further by the nature of the tumor vasculature, which creates additional forms of heterogeneity because of its variable ability (or inability) to deliver adequate nutrients and remove waste throughout a tumor ecosystem. Vascular heterogeneity can create areas of inadequate oxygen (and nutrient) supply leading to intratumoral regions of hypoxia and anoxia, causing affected cells to stop proliferating or, in more extreme cases, die (regions of necrosis). Vascular heterogeneity also can affect drug delivery, with the inability to deliver cytotoxic concentrations of a drug to all tumor cells in a manner that contributes to drug response heterogeneity. Drug response heterogeneity also is complex and can reflect a mix of activities that are intrinsic and/or extrinsic to the cancer cells.

Since it is not possible to cover in depth all forms and mechanisms of heterogeneity, we provide insights mostly from the perspective of responsiveness to therapeutic interventions and the regulation of breast cancer cell fate. For the purposes of this review, we consider cell fate as the balanced outcomes among cell survival-death and proliferation-growth arrest. Readers interested in more in-depth reviews may find the following citations of interest; these address heterogeneity in the context of metastasis (34–36) and the interaction of immune and cancer cells in the TME (37), often from the common perspective of effects on drug responsiveness (38–41).

## Cellular heterogeneity - the tumor microenvironment

One of the clearest examples of cellular heterogeneity is within the TME, which plays a potentially multifaceted role, in collaboration with the extracellular matrix (ECM), in therapy resistance acquisition (42–46). As noted above, the TME comprises a variety of components such as vascular endothelial cells, adipocytes, pericytes, stroma/stem-like cells (MSCs) and extracellular matrix. The TME is subject to dynamic turnover that produces both spatial and temporal intra- and inter-tumor heterogeneity that can directly influence drug responsiveness. In this section, we consider the mechanisms used by TME components to modify endocrine responses in ER+ breast cancer cells.

### Breast cancer-associated fibroblasts

**Figure 2:**
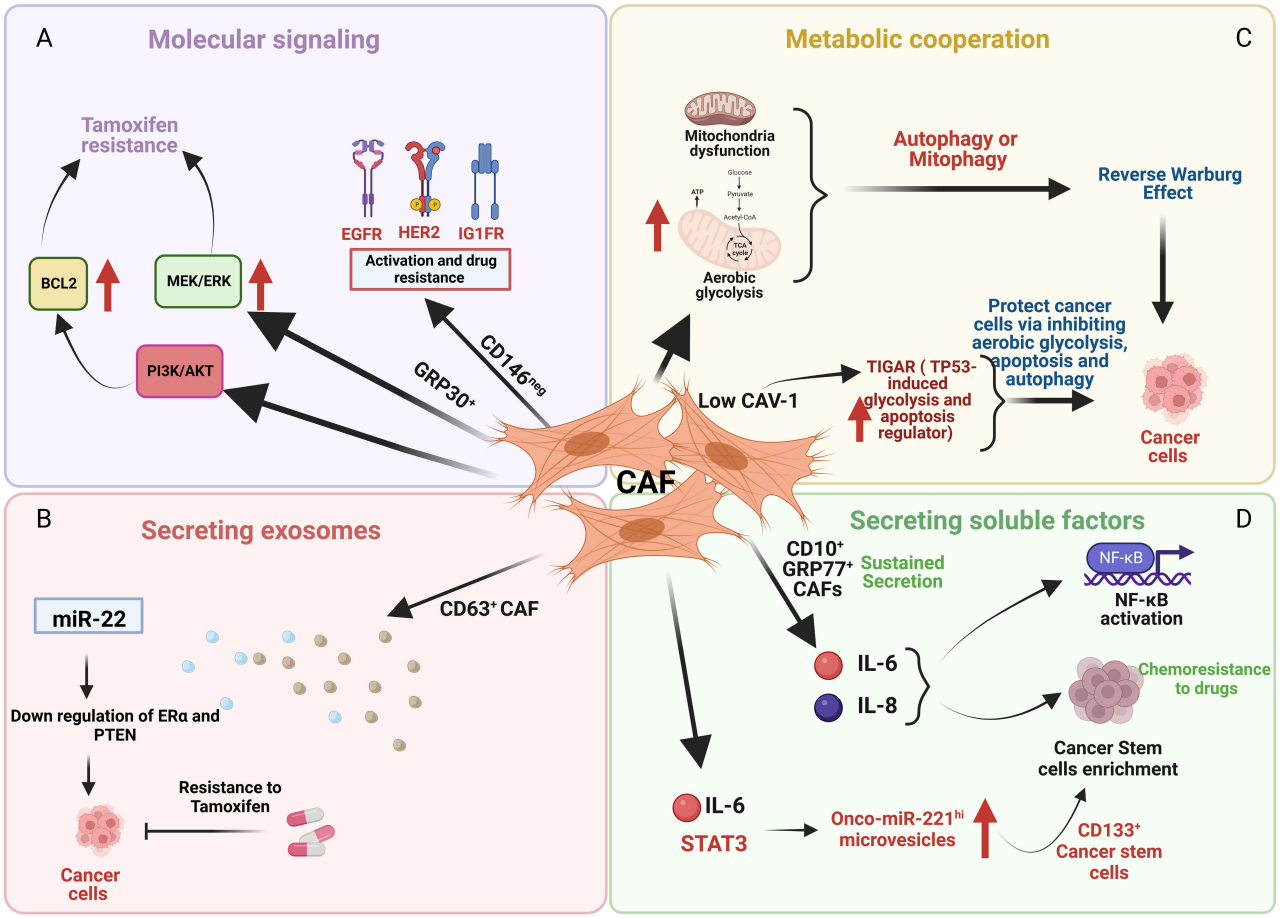
An illustration depicting the effects of cancer associated fibroblasts (CAF) cells on cancer cells through various mechanisms. **(A)** CD146 negative (CD146neg) CAF activate EGFR, HER2, and IGFR in breast cancer cells that promotes tamoxifen resistance whereas GPR30 positive (GPR30+) CAF cells induce tamoxifen resistance by upregulating MEK/ERK and PI3K/AKT signaling. **(B)** CD63 positive (CD63+) CAF promote tamoxifen resistance by downregulating ER and PTEN mediated by exosomes containing miR22. **(C)** Low caveolin-1 (CAV-1) expression in CAF upregulates TIGAR that protects cancer cells by inhibiting aerobic glycolysis, apoptosis and autophagy. **(D)** CD10 positive (CD10+) and GPR77 positive (GPR77+) CAF induces chemoresistance by sustained secretion of IL-6 and IL-8 and enrichment of stromal cancer stem cells.

Cancer-associated fibroblasts (CAF) are among the most important components of the stromal cell population within the TME, and have been shown to participate in many features of cancer progression including altered drug responsiveness (**Figure 2**). CAF subpopulations in breast cancer express different markers such as α-SMA, FAP, PDGFRα, PDGFRβ, CD29, NG2, FSP1, vimentin, PDPN (47–50), CD146 (51) and CAV1 (52). Other markers include the more recently implicated GPR77 and CD10 (53), CX chemokine ligand 12 (CXCL12) (54), MHC-II gene and CD74 (55), Fibulin-1 and SGRG-1 (56); CDK1, CD53 and CRABP1 (55). These markers define heterogeneous subsets of CAFs from different origins (myCAFs, iCAFs, apCAFs, vCAFs, mCAFs and developmental CAFs) that can present different and contrasting roles in breast cancer [for review see (57)], including promotion of chemoresistance or sensitivity. Besides heterogeneity in marker expression, origin and function, the localization of subsets of these cells in the TME also contributes to CAF heterogeneity and can affect their role in cancer progression. Applying simultaneous detection of the vCAF marker Nidogen-2, the mCAF marker PDGFRα, and the dCAF marker SCRG1 identified three distinct stromal populations with divergent growth patterns and localization in relation to the nests of breast tumor cells (56).

### Mechanisms of CAF-induced endocrine resistance in breast cancer

CAFs use different/heterogeneous mechanisms to drive ER+ breast cancer cells to acquire endocrine resistance. Mechanisms of the activated TME that can induce therapy resistance include promotion of pro-survival pathways, stemness traits, and/or metabolic reprogramming; CAFs can regulate each of these mechanisms to induce endocrine resistance in ER+ breast cancer cells.

Examples of different CAF subsets regulating several pro-survival mechanisms are evident. For example, CD146 expression defines two subsets of CAFs, CD146-positive (CD146^pos^) and CD146-negative (CD146^neg^) in luminal breast cancer (**Figure 2A**) (51). In these breast tumors, CD146^neg^ fibroblasts exhibit decreased ER expression, whereas ER^+^/CD146^pos^ fibroblasts remained estrogen responsive and antiestrogen sensitive. Breast cancer cells influenced by CD146^neg^ fibroblasts may escape estrogen-dependent proliferation and exhibit tamoxifen resistance through activation of EGFR, HER2, and IGF1R (51). Weigel et al. (2012) implicated a role for enhanced platelet-derived growth factor receptor (PDGF)/Abl signaling in aromatase inhibitor-resistant breast cancers (58). Both tumor and fibroblast expression of PDGFRα and PDGFRβ was significantly correlated in pre-treatment and relapse samples and high post-treatment tumor and fibroblast PDGFRβ levels were associated with a short time to treatment failure (TTF).

GPR30 (G-protein receptor 30)-expressing CAFs induce tamoxifen resistance involving MEK/ERK signaling in ERα-positive breast cancer cells and tumors (**Figure 2A**) (59). CD63^+^ CAFs may promote tamoxifen resistance by secreting exosomes rich in miR-22, which can induce downregulation of ER and PTEN, to confer tamoxifen resistance on breast cancer cells (**Figure 2B**) (60).

Several other studies reported the induction of pro-survival signaling through PI3K/AKT and MEK/ERK by CAFs to promote endocrine resistance (61–63). For example, activation of the PI3K/AKT pathway that phosphorylates S-167 of ER increased BCL2 expression and altered resistance to tamoxifen following activation of fibroblast growth factor receptor 2 (FGFR2). These activities reversed tamoxifen-driven ER stabilization and promoted ER phosphorylation and proteasomal degradation (61).

Activated signaling through PI3K/AKT/mTOR is often driven by mutations in either AKT and/or PIK3CA (64–66) and is clinically actionable with drugs that target each of these signaling nodes. Several clinical trials with these drugs have been completed and others are ongoing; none has yet produced any notable increase in OS. While some studies show improvements in the clinical benefit ratio, these are often associated with significant dose limiting toxicities (see (67) for review). A major challenge is feed-back activation within the signaling feature when one node is inhibited, making shutting down the signaling more difficult. While novel drug combinations and scheduling could mitigate some of this feedback activation, these are likely also to be accompanied by significant toxicity for patients. Moreover, the potential of cellular or molecular heterogeneity to further limit responsiveness is likely to remain a challenge. While patients can be selected for specific drugs based on the AKT and/or PIK3CA mutational profile of their tumor, it remains difficult to predict which tumors are driven by the detected mutation, perhaps reflecting the difficulty in predicting the true penetrance of the mutation (where the presence of the mutation is responsible for the observed tumor phenotype).

Examples of metabolic cooperation between CAFs and tumor cells that can alter endocrine responsiveness include the loss of stromal caveolin-1 (Cav-1), a predictive marker of poor clinical outcome in breast cancer patients treated with tamoxifen (68). Martinez-Outschoorn et al. showed that mitochondrial dysfunction, oxidative stress and aerobic glycolysis are increased in CAV1-downregulated CAFs (69). The authors proposed that defective mitochondria are removed from these cells by the autophagy/mitophagy induced by oxidative stress. These autophagic processes could provide nutrients (such as lactate) to stimulate mitochondrial biogenesis and oxidative metabolism in adjacent cancer cells. Martinez-Outschoorn et al. showed that low CAV-1 CAFs, after treatment with tamoxifen, increased TIGAR (TP53-induced glycolysis and apoptosis regulator) expression (**Figure 2C**) (70). TIGAR is a p53-regulated gene that simultaneously inhibits glycolysis, autophagy and apoptosis and decreases ROS, thereby promoting oxidative mitochondrial metabolism in ER+ breast cancer cells. CAFs showed increased glucose uptake and glycolytic activity, impaired mitochondrial function, and increased generation of lactate and ketone bodies; conversely, epithelial cancer cells in coculture display increased mitochondrial activity and protection from apoptosis (70).

CAF subpopulations can promote breast cancer stemness and treatment resistance including resistance to endocrine therapies. CD10^+^ GPR77^+^ CAFs induced chemoresistance *via* sustained secretion of IL-6 and IL-8 (**Figure 2D**) (53). Sansone et al. determined that the IL6-pStat3 pathway (required for CAF proliferation) promoted the biogenesis of onco-miR-221^hi^ CAF microvesicles (MV) which, in combination with hormone therapy, established stromal cancer stem cell (CSC) niches, specifically those with CD133^hi^ cells (**Figure 2D**) (71).

Targeting CAF heterogeneity and their different molecular mechanisms could prevent or reverse resistance to endocrine therapies and suggests one approach for the development of more effective therapeutic interventions.

## Tumor associated macrophages

Macrophages are abundant in the TME (72). The role(s) of tumor-associated macrophages (TAM) in the pathogenesis of cancer depend on their phenotype and polarization (31), which can be affected by the TME (73, 74). The importance of TAM localization within different tumor compartments has been reviewed by others (31). TAM phenotypes are often characterized as either M1, which promotes proinflammatory and tumoricidal responses, or M2, with anti-inflammatory and pro-tumor responses (32). Markers of M1 TAM include HLA-DR and CD80/86; M2 markers include CD206, CD163, CD204, and stabilin-1. In breast cancer, the M2 markers CD47, COX-2, MMP9, TIE2, YKL-39, YKL-40, PD-L1 were also identified (31). TAMs can play a role in endocrine resistance; for example, macrophages may induce endocrine resistance in ER+ breast cancer cells by inducing sustained release of TNF-α and IL-6 from breast cancer cells, resulting in activation of NFκB, STAT3, and ERK-1 and hyperphosphorylation of ERα (75). Formation of an NFκB/STAT3/phospho-ER complex in cyclin D1 gene correlated with increased proliferation, independent of ER ligand status (75).

## Breast cancer stem cells

Cancer stem cells are a population of tumor cells with the capacity to self-renew and to generate more stem cells and also to differentiate into other cell types (76). Breast cancer stem cells contribute to breast cancer heterogeneity, expressing a highly diverse profile of markers in different tumor subtypes ( (77) for Review). Markers reported to be associated with the ER-positive, Luminal A and/or Luminal B, subtype include: CD44+/CD24-/low, MUC1+/CD24+, CD44+/Vimentin+, CD44+/Osteonectin+, CD24+/CK18+, CD24+/GATA+ (50). The marker ALDH1 has the highest expression in HER2+ and basal-like tumors (77).

While playing a role in breast cancer initiation and growth, evidence of the role of breast cancer stem cells in metastasis (78) and therapeutic resistance (79) has also been reported. Simoes et al. showed that both tamoxifen and fulvestrant induce STAT3 phosphorylation and activation of the STAT3 target genes MUC1 and OSMR in ALDH+ cells from endocrine-resistant patient samples (80). The SOX2 pathway, which is associated with stem cell characteristics and involved in embryonic development (81), and the Wnt pathway that is associated with the epithelial to mesenchymal transition (82), were both activated after endocrine treatment of ER+ breast cancer cells. Activation was accompanied by an increase in CD24 but not CD44 expression. Together, these observations suggest an early adaptation to endocrine stress with increased stemness that enables the survival of emerging hormone-resistant cell populations (83).

The Notch pathway can regulate breast cancer stem cell activity (84, 85) and has been implicated in endocrine therapy resistance (86–88). FK506-binding protein like (FKBPL), an anti-tumour protein that belongs to the family of immunophilins, was shown to inhibit endocrine therapy resistant CD44+ cancer stem cells in ER+ disease, *via* modulation of the components of the Notch pathway, DLL4 and Notch4 (89). Liu et al. showed that everolimus, a mTOR antagonist, in combination with letrozole inhibited MCF-7 ESA(+)CD44(+)CD24(-/low) stem cells *via* PI3K/mTOR signaling (78).

## Molecular heterogeneity

The evolution of clonal tumor cell populations alters intratumor heterogeneity spatially and/or temporally. Earlier studies have indicated intratumoral heterogeneity of breast cancer biomarkers, such as ER, PR, and HER2 (90–92). Studies using large scale genome analysis have mapped the complex mutational landscape and confirmed the presence of genetic clonal subpopulations in breast tumors (93–98). Typically, single biopsies are used to determine the features of a tumor. However, single biopsies are temporally and spatially restricted because the cells collected often represent a small fraction of the tumor composition present at a single time point. Therefore, a biopsy may not capture the entire diversity of tumor cell populations and can fail to reflect fully spatial heterogeneity. For example, two different tumor cell populations can exist spatially as ‘collision tumors’; these may only be observed when the entire tumor is analyzed, such as after surgical resection. Indeed, when the clonal architecture of cancer cells was monitored after four months of neoadjuvant aromatase inhibitor therapy, tumors of independent origin were found that were not captured in the baseline biopsy (99).

One approach to overcome this challenge is to perform multiregion sequencing of biopsies from the same tumor. For example, multiregion sequencing from 8 biopsies of single treatment naïve breast tumor found subclonal diversification and geographically constrained patterns of subclonal growth leading to extensive and statistically significant spatial heterogeneity of point mutations (100). Due to geographically restricted subclonal growth, in 4 cancers (out of 12), the detected driver mutation was present only in 1-3 samples from 8 needle biopsies (100). The challenge of both spatial and molecular heterogeneity in subpopulations requires careful consideration when searching for newly detected mutations after treatment, especially if a single needle biopsy was used to determine heterogeneity at baseline. Recent studies have confirmed that most ductal carcinoma *in situ* (DCIS) lesions that give rise to recurrent invasive breast cancer share some clonal relationship with the initial DCIS (101). Any treatment that fails to eliminate all driver clones in DCIS will likely lead to recurrence, independent of how effective the treatment may appear when tumor shrinkage is used as the marker of response. Normal mammary cells associated with tumors in breast cancer patients may also harbor pathogenic variants of p53 and PIK3CA (102), suggesting that clonal (and genetic/molecular) heterogeneity may be a fundamental feature of mammary cells.

## Genetic heterogeneity

Cancer conforms to evolutionary rules defined by the rates at which clones mutate, adapt, and grow. Acquired gene mutations are inherent for cancer cells, where some gene mutations are defined as driver mutations that have known functions capable of conferring survival and proliferation advantages (103, 104). Tumors contains different subpopulations of cells (subclones) that can be distinguished based on different characteristics affecting their phenotype including genetic alterations such as single-nucleotide variants (SNVs), small insertions or deletions (indels), somatic copy number alterations (CNAs), and structural variants. Chromosomal and genomic alterations have been documented in most cancers including breast cancers (98, 105, 106), some of which clearly affect biological processes and functions (107) that may facilitate acquired resistance to specific therapeutic interventions (108). Analysis of the whole genome sequences of 560 breast cancer patients revealed 93 protein-coding genes that contained probable driver mutations (109). The ten most frequently mutated driver genes accounted for 62% of all driver genes and included TP53, PTEN, ERBB2, FGFR1 locus, GATA3, RB1, and MAP3K1. TP53, RB1, and PTEN were more frequently found in ER negative breast cancers. PIK3CA, CCND1, and FGR1 mutations were more frequently detected in ER+ breast cancers (109). Other studies have also reported alterations in these genes in breast cancer (98, 110). Metastatic breast cancers frequently share the majority of their genomic alterations with the corresponding primary disease, indicating pre-existing clones. However, metastases at distant sites also continue to acquire new mutations that were not previously detected or are subclonal in the primary disease (110, 111). For example, ESR1 activating mutations are rarely present in primary ER+ breast cancer, even in those 15–20% of patients that show intrinsic resistance to hormonal therapies (112). However, these mutations are enriched in ER+ metastatic tumor samples from patients with acquired endocrine resistance (30–40% of ER+ patients), mostly following failure of an aromatase inhibitor-based intervention (113). International Cancer Genome Consortium (ICGC) and Cancer Genome Atlas (TCGA) have thoroughly explored the genetics of primary breast cancer and sequencing of breast cancer samples. These studies revealed nine cancer genes (TP53, ESR1, GATA3, KMT2C, NCOR1, AKT1, NF1, RIC8A and RB1) to be highly mutated in the metastatic disease when compared with early breast cancer (114). Importantly, genomic comparisons of primary and metastatic samples showed that metastatic breast tumors frequently possess higher numbers of mutations (mutational load), including driver mutations and somatic copy number aberrations, than matched primary tumors (115). Some of these mutations are associated with endocrine therapy response (110, 116, 117). For instance, the prevalence of activating ERBB2 mutations and NF1 loss of function increased two-fold in endocrine resistant tumors; regulators of ER mediated transcription were also enriched in endocrine therapy resistant tumors (117).

## Transcriptional heterogeneity

Transcriptional heterogeneity plays a key role in the development of different cell states. Different transcriptional programs contribute to dynamic plasticity and allow co-existence of tumor cells with differential drug sensitivities that may support the development of resistance to specific therapies (118). Earlier studies in lung cancer cells showed that in a drug sensitive cell population a small proportion of cells are reversibly drug resistant because of chromatin mediated signaling (119). Using single cell RNA-seq analysis, the transcriptomic heterogeneity of different type of cells in tumors has been cataloged (120). Profiling tumors from 11 breast cancer patients using single cell RNA-seq reported both common and diverse transcript signatures from cancer cells of the same tumor (121), whereas most non-cancer cells were immune cells. This study further revealed that in the ER+/HER2+ breast cancer subtype, the primary tumors showed predominant ER signaling but cells from the metastatic lymph nodes expressed mostly HER2-activated genes. Conversely, neoadjuvant treatment of HER2+ breast cancer changed the gene expression profile of the tumor to a pattern that resembled triple negative breast cancer (121). Thus, both disease progression and treatment can contribute to temporal transcriptional heterogeneity.

Single cell studies have revealed that transcript heterogeneity also exists in commonly used breast cancer cell lines. In MCF7 cells, intracellular transcriptomic heterogeneity was dominated by cell cycle states but a rare subpopulation of MCF7 cells showed a unique transcriptional feature with an apoptotic signature (122). Using single cell transcriptomics, spatially resolved data identified cell modules with different activated pathways in a single tumor. Pockets of basal neoplastic cells in tumors were detected in tumors characterized as ER+ positive breast cancer with bulk sequencing (123). In addition, a lobular carcinoma with low cellularity for neoplastic cells was misassigned as ‘normal-like’ by bulk sequencing (123).

Extensive transcriptional heterogeneity was reported in triple negative breast cancers (TNBC), with certain subpopulation of cells showing a highly malignant gene signature (124). A minor breast cancer cell population with mesenchymal properties could immunosuppress the TME and help the entire tumor to evade immunotherapy (125). Existence of subclonal populations in a primary TNBC tumor may be responsible for metastatic dissemination and seeding of cells in distant organs (126). Using primary and its paired metastatic cells coupled with single cell sequencing of three TNBC patient derived xenografts, metastatic cells were found to be proficient in oxidative phosphorylation, whereas its matched primary tumor cells were dependent on glycolysis (127).

High intercell variability at the transcript level may be responsible for metastasis and resistance to chemotherapy that can be attributed to differences in splicing machinery (128). In ER+ breast cancers, development of resistance to endocrine therapy was found to be gradual and required multistep adaptation (129). Using single cell methods the pre-existence of therapy resistance subclones defined by distinct transcript profile of single cells may be unlikely (129). In a recent report that studied serial biopsies using single cell RNA sequencing from early stage ER+ breast cancer tumors treated with AI therapy plus CDK4/6 inhibitors, results revealed an emergence of common resistant phenotype that showed loss of estrogen signaling and upregulation JNK signaling (130).

While the use of single cell RNA sequencing has begun to offer new insights into cellular and molecular heterogeneity, many studies inadequately consider the limitations of current technologies. Few methods are yet able to sample more than ~50% of the entire transcriptome, leaving many RNA species undetected. Leveraging bulk sequencing data on the same samples may increase the ability to see further into the transcriptome. While this limitation is clearly problematic for studies into mechanism, it is less challenging for biomarker and similar studies where the entire transcriptome does not need to be sampled adequately.

## Metabolic heterogeneity

Metabolic reprogramming is a hallmark of cancers that progress (131, 132) and drives resistance to therapies including endocrine-based interventions (133). However, all tumors or tumor cells may not show the same metabolic adaptations and may exhibit heterogeneous metabolic phenotypes and metabolic plasticity (134, 135). For example, different anatomic sites show different metabolic dependencies. Glucose oxidation was dominant in brain and lung tumors, but glycolytic intermediates were elevated in renal cancer cells, where glucose oxidation was suppressed (136–138). Conversely, different subtypes of breast cancer show different dependencies for glutamine. Generally, luminal breast cancer cells can resist glutamine deprivation but basal cells are dependent on external glutamine (139). However, glutamine is required for proliferation in advanced HR+ breast cancer cells (140, 141). Differences in nutrient and oxygen availabilities impacts metabolic dependencies. Vascular heterogeneity causes oxygen gradients in tumors that can render the environment hypoxic for those tumor cells distant from blood vessels (142). Hypoxic conditions activate hypoxia inducible factors (HIFs), while in the presence of sufficient oxygen the proto-oncogene c-Myc may drive a transcriptional program that promotes uptake of nutrients and increases the rate of glycolysis (142). Indeed, c-Myc is involved in glucose and glutamine uptake in endocrine therapy resistant cells and promote cell proliferation (143, 144). HIF1-alpha is also involved in endocrine therapy resistance by stimulating the expression of various glycolytic enzymes in endocrine therapy resistance (145–148).

## Drug response heterogeneity

The ability of a systemic therapy to eradicate a tumor and its metastases is largely dependent on the ability to overcome the heterogeneity of responses in individual cells. Drug response heterogeneity is multifactorial and reflects the combined effects of many of the other forms of heterogeneity discussed above. Cytotoxic drugs generally target cellular features or functions associated with replication, *e.g.*, DNA synthesis (drugs that include alkylating agents, antimetabolites, anthracyclines) or the dynamics of microtubule turnover and their function during mitosis (vinca alkaloids destabilize and taxanes stabilize microtubule dynamics).

Cancer cell replication in solid tumors is asynchronous, with many cells arrested in G_0_/G_1_ and so insensitive to drugs that target the replication machinery while the cells remain growth arrested. This form of resistance is often referred to as ‘kinetic resistance’. Some cells may remain arrested for a sufficient period that the drug target is released from its inhibition before the cells receive and respond to a mitogenic stimulus. Cells can then enter S-phase with the ability to complete the cell cycle and continue to proliferate.

Endocrine therapies are also affected by kinetic resistance, although the response pattern can differ from that seen with cytotoxic drugs. In ER+ breast tumors, a drop in proliferation as induced by an endocrine therapy and reflected by an often dramatic reduction in Ki67 expression (149, 150), can predict a good clinical response (151). Since endocrine therapies are usually given daily for 5-10 years (152), the growth inhibitory activities that arrested cells into G_1_ may further drive them into G_0_, effectively taking them out of the cell cycle for prolonged periods. This prolonged growth arrest may allow cells that do not undergo cell death in response to the stress of an endocrine therapy to rewire their signaling and cellular functions to ensure that they survive but remain growth inhibited (23). Rewiring is likely epigenetically maintained (and so reversible) enabling cells to re-enter the cell cycle when conditions are favorable and appropriate mitogenic signals are received and executed. The dormancy strongly associated with ER+ breast cancer (153–155) is one reflection of these types of events, as is the response reported to some therapeutic regimens that include an inhibitor of enzymes that induce and maintain epigenetic modifications (156, 157).

The molecular and genetic heterogeneity within cancer cells also contributes to drug response heterogeneity. For example, not all cells in an ER+/HER2+ tumor concurrently express both functional ER and HER2, yet drugs that target each can induce significant clinical benefit. Nonetheless, all ER+/HER2+ tumors are not cured with combined anti-ER and anti-HER2 interventions and there can be some cells in these tumors that may express neither ER nor HER2. Genetic heterogeneity, when applied to an actionable mutation in genes such as PIK3CA (Alpelisib) or AKT (Capivasertib), likely contributes at least partly to the inability of drugs targeting these mutations to yet show a major improvement in overall survival (158–162). Since not all cells express the mutated gene, and some may express the mutation but are no longer driven by its signaling, many tumors may exhibit a phenotype where the penetrance of the mutation may appear low. For example, some tumors with a PIK3CA mutation many not exhibit endocrine resistance and/or respond to a PIK3CA inhibitor.

One of these drug response modifying features is the vascular heterogeneity that is often a significant feature of breast and other solid tumors. Systemically administered drugs must reach the cells that express their molecular targets, and do so in sufficient concentrations to kill the cancer cells. Vascular heterogeneity can lead to different perfusion gradients in tumors, where some regions may receive a cytotoxic level of a drug and others do not - also creating a form of spatial heterogeneity. Vascular heterogeneity may induce a multiple drug resistant-like phenotype since inadequate vascularity may prevent many drugs, independent of their chemical structure or mechanism of action, from reaching some areas of a tumor. Regions that are poorly vascularized may also be hypoxic, with induction of hypoxia stress responses already active in surviving cells in a manner that may also confer resistance to some drugs that reach these hypoxic cells (163–165).

Cellular heterogeneity within the TME likely also contributes to drug response heterogeneity. The presence of multiple interacting cell types can alter the drug responsiveness though their ability to communicate and modify cellular and molecular functions. Among the more common mechanisms are paracrine (such as the secretion of growth factors (166–168) or microvesicles (169–172) and juxtacrine (often through gap junctional intercellular communication) (173, 174). Thus, cellular heterogeneity can affect molecular heterogeneity and so also drug response heterogeneity.

Spatial heterogeneity also contributes to drug response heterogeneity. At the macrolevel, different metastatic sites in the same patient may respond differently to a systemic therapy. For example, brain metastases can be difficult to manage because the blood-brain barrier can prevent adequate concentrations of many drugs from reaching brain lesions. It is likely that vascular heterogeneity further compounds this issue, since it can confer some degree of spatial heterogeneity at the microlevel (within a single tumor) as noted above for areas of hypoxia.

The combined features of cellular, molecular, vascular and spatial heterogeneity are often further modified by temporal heterogeneity. For example, many omics data are collected as massively parallel snapshots of the cell population samples (175). Tumor cells and the TME are often in a dynamic state. Tumor cells are often genetically unstable and acquire additional mutations over time. As many solid tumor progress, genetic heterogeneity increases. Since the length of time for the acquisition of new mutations and/or other molecular features differs in cells, we face another temporal feature – biological time. Some breast tumors may grow rapidly, others remain indolent and may grow very slowly. Acquiring new mutations is often a consequence of failure to recognize and repair errors in replication. The probability of acquiring a new uncorrected error is related to the frequency of DNA replication, and so is expected to be greater in rapidly proliferating than growth arrested cells. Hence, differences in biological time, such as the time it takes to acquire new driver mutations, can create spatial heterogeneity (some areas of a tumor or some metastases in a host may grow at different rates and experience differences in biological time). Since biological time is related to the acquisition and loss of biological features/functions, biological time can also create spatial, temporal, genetic, and molecular heterogeneity.

Changes in the TME, including the dynamic influx and activation/inactivation of key immune effector cells, ensure that interactions between the TME and the tumor cell compartment are constantly changing. Over time, some cells acquire the ability to invade locally and eventually leave the primary tumor to seed metastases, such that the genetic, molecular and cellular features of the tumor also change. Each of these, whether alone and/or in combination, may affect the response of a tumor to a drug and may do so differently over time. In this example, time can be temporal or biological, reflecting the evolution of the tumor and its associated TME. Application of a drug can further change these features over time. For example, breast tumors with many growth arrested cells due to an endocrine intervention may be relatively unresponsive due to kinetic resistance. However, once these cells escape inhibition, perhaps with a wave of proliferating cells driving rapid emergence from dormancy (no longer exhibiting kinetic resistance), the same cells may be more responsive to drugs that require cells to be progressing through the cell cycle to be effective.

Pharmacogenetic differences, reflecting the differential expression of isoforms of genes that affect drug metabolism, also can affect drug response heterogeneity. This form of heterogeneity is most evident at the population level, and among individual patients rather than within a tumor and its TME (176–178). Other factors that differ among patients and affect the pharmacokinetics or pharmacodynamics of a drug can also produce drug response heterogeneity but are beyond the scope of this review (179, 180).

## Concluding comments and future directions

We have described several of the key forms of heterogeneity and some of their interactions and modifying factors. For example, genetic heterogeneity is one driver of molecular heterogeneity and cellular heterogeneity is one reflection of molecular heterogeneity. A focus on cellular heterogeneity has become common in recent years. However, this seems likely to change as the multifaceted nature of heterogeneity becomes more widely appreciated and studied. Advances in spatial omics, such as single cell DNA and RNA sequencing, are likely to change our understanding of many forms of heterogeneity and their interactions. Nonetheless, current limitations in these new methods are evident, as is often true when a new technology emerges. For example, the depth of omics coverage can be limited for each single cell. In a transcriptome of perhaps 50,000 different transcripts, less than 50% may be detected, unambiguous assignment of sequences to their correct mRNA can be difficult, and noise in the data may be quite high (181, 182). The number of cells that can be studied is limited, often to a few hundred or thousand cell in a tumor cell population of millions (a clinically palpable 1 cm tumor has ~10^9^ cells) (183). All of these cells concurrently reflect the effects of cellular, molecular, genetic, spatial, and temporal heterogeneity in ways that may not be reflected in the population sample as analyzed. Single cell proteomics and metabolomics face similar challenges, where depth, coverage and the ability to unambiguously assign a signal to a single molecular entity may be no better, or perhaps lower, with current technologies (184–186).

Current spatial omics technologies represent only one series of tools with which to explore the complexity of heterogeneity. Given the rapidity of technological advances in general, it is hoped that the current limitations with these tools will soon be overcome. It will be particularly important to constrain the cost and maximize the availability of these and other new technologies not yet brought to market, to allow the full breadth of the research community to have access and so more effectively advance knowledge.

## Author contributions

All authors contributed to the writing and editing of this review. All authors contributed to the article and approved the submitted version.
